# A Recent Epidemic of Scabies

**Published:** 1904-12

**Authors:** E. Wood Ruggles

**Affiliations:** Rochester, N. Y.


### A Recent Epidemic of Scabies.[1]

By E. WOOD RUGGLES, A. M., M. D., Rochester, N. Y.

SCABIES is generally termed a filth disease. In the majority of cases it is confined to the foreign, thickly housed and dirty classes and its prevalence is far greater in Scotland and continental Europe than in the United States. Its frequency has been pretty steadily increasing of late years, however, since the character of the immigration to the United States has changed and the great majority of our new citizens come from Italy, Russian and Austrian Poland, Roumania and Hungary, with a large admixture of the still filthier classes from the Orient. The intimacy of life in the steerage, with several occupying one bunk, infects a great many who were free from the disease when they sailed. The medical inspection of immigrants should exclude these persons, but it does not.

The "day nurseries" in large cities, where children are cared for during the absence of their mothers at work and are returned home at nightfall and the various "homes" and "asylums" for children serve to propagate the disease in many families. The bedding in hotels, and sleeping cars, which is rarely completely changed when the bed has been occupied but one night, is a prolific source of the disease. Hotel keepers in the state of Ohio seem to be particularly bad offenders in this respect, judging from the fact that apparently the only possible source of contagion in several of my own and Dr. Grover W. Wende's cases was a sojourn of some days in Ohio hotels, without other residence aside from their own homes.

Domestic animals cause a few cases, but their parasite is not long lived when transferred to the human race. Finally, much spreading of the disease is caused by failure in its diagnosis. I have had referred to me within the past ten days by a first-class physician a young man covered with vesicles, pustules and furuncles, caused by the disease, whom he had been treating with soothing ointments and letting sleep with his brother.

It is very interesting to observe the increase in the percentage of cases of scabies to the whole number of skin diseases treated in various dispensaries. In all of the dispensaries of Philadelphia this percentage increased from 1 2-3 per cent. in 1880 to 14½ per cent. in 1891 and 13½ per cent. in 1892. In all the dispensaries of five cities, New York, Boston, Baltimore, Chicago and St. Louis, it increased from 9-10 of 1 per cent. in 1880 to an average of 6 per cent. from 1887 to 1891 inclusive.

---

1. Read at the annual meeting of the Medical Society of the County of Monroe, at Rochester, N. Y., May 25, 1904.

In private practice also the percentage has increased considerably, if more slowly. Of the cases of skin diseases reported by all the members of the American Dermatological Association, it increased from ½ of 1 per cent. of all cases in 1880 to 2 17-100 per cent. in 1887. Stelwagon says he never saw a case in private practice prior to 1886 or 1887, while he now sees a score or more yearly. Having lost my records by fire it is difficult to state just how many cases I have myself seen during the past year, but at least 10 or 12. Have seen two during the past three weeks.

There also occur epidemics of the disease, as for instance during the Chicago exposition, when there was such a large influx of low class foreigners, especially from the Orient. The percentage of cases in Chicago doubled at that time and the St. Louis exposition will undoubtedly produce the same result. At such times the disease invades the well-to-do classes to a much greater extent than otherwise. During the past year there has been such an outbreak in Rochester and vicinity though I have seen no statement that it was general. In my own practice there have been at least three times as many cases of itch in proportion as during any other year and several physicians in general practice have told me the same.

Dr. Wende informs me that the same condition obtains in Buffalo, his proportion of cases having been much larger during the past year.

The Spanish-American war during which so many young men of the better classes went to Cuba as soldiers and the subsequent greatly improved relations between this country and Cuba, resulting in much visiting back and forth, is most probably the cause of this last epidemic, scabies being very prevalent on the island. The return of many soldiers and other visitors to the island infected with the disease, to their own homes, would account for an unusual feature of the epidemic—namely, its prevalence among the well-to-do, the majority of my cases having occurred in families where one would least expect it.

In these cases also the disease has, as a rule, presented very poorly marked features. There were few or none at all of the characteristic burrows or cuniculi and the disease could only be recognised by the situation of the affected areas and by the presence of an eczema which was evidently not idiopathic, but produced entirely by scratching. The pruritus which causes this, is often out of all proportion to the discoverable lesions which, in those who bathe frequently are, as a rule, very insignificant, consisting of isolated scratch marks, small papules, occasionally a row of minute pustules. Of course, many cases of simple pruritus also present few or no lesions and the locality and multiformity of the lesions are the chief guides to diagnosis.

On close examination I have nearly always found some burrows, excoriations, papules, vesicles, pustules or crusts present in the intradigital spaces of hands or feet and, as a rule, on the glans and shaft of the penis. Next in frequency are the flexor surfaces of wrists, buttocks and anal region, scrotum, axillæ, abdomen, breasts of women and any area where pressure produces warmth, such as under the waistband of trousers or under corsets.

The burrow rarely assumes its classical appearance and the textbook writers must possess vivid imaginations to describe it as "a whitish or a yellowish, slightly arciform, linear lesion with regular parallel borders, covered with dots or specks representing the feces of the mite." Occasionally, one may meet such a lesion, but I am sure I have never yet found one corresponding to all these details.

It is also comparatively easy, according to the books, to isolate the acarus for microscopical examination, but I have rarely seen it done, and felt much satisfaction on securing a perfect specimen last December. These mites are very minute, only 1-100 to 1-70 of an inch in their long diameter and are, as a rule, completely concealed by the drop of blood, serum or pus liberated through the operation for their attempted extrication. Diagnosis therefore is generally a matter of impression rather than of absolute certainty. But in the presence of an obstinate pruritus, associated with fingernail marks, indicating the great severity of the itching, and multiform eczematoid lesions, especially in a young and otherwise healthy individual, in whom we would not await a pruritus *per se,* it is well to always have scabies in mind, to strip the patient and notice the regions particularly affected. Then by close examination we may find a few burrows, perhaps one only, marked by a row of tiny vesicles or pustules diminishing in size toward one end, sometimes a pustular tract like that left by withdrawing a splinter which has remained under the skin a few days. We shall surely find a certain linear character to some of the lesions, that whether papules, vesicles or pustules, they are longer in one diameter and oval or oblong in shape. In a few cases the black specks, representing the feces and looking something like black-heads, only that they are looser and more easily removed, are found.

Such lesions when found scattered over different parts of the body, especially when multiform in character, excoriations, papules, vesicles, perhaps pustules occurring simultaneously and principally on fingers, hands, wrists, axillæ, breasts in women and the penis should be diagnosed as scabies, although no acari are found, and treated as such. The treatment can do no harm if the diagnosis is a mistaken one, while failure to cure a case not only prolongs the misery of the patient, but spreads the disease.

As to treatment there are two essentials; first, to kill the parasites, not only in the skin, but in the clothing and bedding also, by moist or dry heat, and, second, to follow up the case long enough to be sure it is cured. Sherwell's washed sulphur treatment, rubbing the patient very thoroughly with the dry powder after a prolonged hot soap bath and spreading the powder on the lower bed sheet has failed me but once, and it is the only cleanly method. Precipitated sulphur, 1 part to 5 or 6 parts ointment is very effective. Kaposi's ointment:

| | | |
|---|---|---|
| ℞ | Naphthol | 25 parts. |
| | Green soap | 50 " |
| | Creta alba | 10 " |
| | Benzoated lard | 100 " |

And Stelwagon's

| | | |
|---|---|---|
| ℞ | Sublimed sulphur, balsam Peru, āā | ℥ j. |
| | Naphthol | ʒ ½ to 1. |
| | Benzoated lard or Ung. petrolati } q. s., ad | ℥ iv. |

are among the very best prescriptions. Two or three applications often suffice to kill the parasites, but the accompanying eczema, often aggravated by the treatment, may require some time for its cure.

On handing in the title of this paper, I had several interesting cases to report, but my records having been burnt, I can give the details of only three recent cases.

M. B., 28 years old, fine family, came to me last July for an intolerable pruritus. His arms were hyperemic and swollen from constant rubbing, but he had practically no characteristic signs of scabies. I believed it to be such, however, and put him on the washed sulphur dry treatment. He gave it a fair trial and getting little relief consulted one or two other physicians and several drug clerks, getting a different diagnosis and treatment from each. In December he returned to me and at this time I found one fairly characteristic lesion, from which I extricated a fine specimen of the female acarus, with a ripe ovum. Under more vigorous treatment he was entirely cured in two or two and a half weeks, as well as his brother who had contracted the disease from him.

Dr. X., a young dentist, consulted me in January for a very obstinate pustular dermatitis for which two physicians had been treating him for eczema several weeks. The pustules on and between the fingers and especially on the wrists were so deep-seated and painful that he had been forced to give up practice. He could not draw his coat on or off without excruciating agony and even with help it was a long and painful task. To complicate matters he had a painful muscular affection of the arms, which at first seemed caused by infection from the very deep and virulent scabies pustules. It proved, however, that he had a urethral discharge containing gonococci and a posterior urethritis, though he had been discharged as cured. The gonorrheal rheumatism as well as the urethritis yielded to appropriate treatment and the scabies and resulting eczema, though deep-seated and obstinate, were cured in about three weeks.

C. K., 25 years old. Acquired scabies some time in November last. While there were some lesions on the penis, he performed coitus, about December 1. He saw Dr. Ernest Wende, at Buffalo, about Christmas, who diagnosticated scabies with particular involvement of the penis. The lesions, except on the penis, healed very satisfactorily, and Dr. Wende prescribed another and much thicker ointment for that region. The glans penis then became increasingly inflamed and tender, urination being exquisitely painful. At the time I first saw him, January 15, the glans penis was intensely congested, there were three denuded areas, about pea-size, one of them surrounding and extending into the meatus. His face showed the intense pain he was constantly suffering. The condition seemed to be the result of over-treatment, but I called his attention to a loss of substance on one lip of the meatus, and told him it might prove to be a case of syphilis. The general inflammation yielded slowly to alum acetate solution followed by an ointment of equal parts of emplastrum plumbi and olive oil, but I was obliged to cauterise the denuded areas to hasten repair. About two and a half weeks later, I found a roseola present and this with the glandular enlargement, which had been slowly progressing, confirmed my diagnosis of syphilis. Upon beginning active syphilitic treatment, with the use of an ointment of white precipitate locally, healing was immediate.

294 ALEXANDER STREET.

